# Gene-gene interactions among coding genes of iron-homeostasis proteins and *APOE*-alleles in cognitive impairment diseases

**DOI:** 10.1371/journal.pone.0193867

**Published:** 2018-03-08

**Authors:** Veronica Tisato, Giovanni Zuliani, Marco Vigliano, Giovanna Longo, Eugenia Franchini, Paola Secchiero, Giorgio Zauli, Elvezia Maria Paraboschi, Ajay Vikram Singh, Maria Luisa Serino, Beatrice Ortolani, Amedeo Zurlo, Cristina Bosi, Antonio Greco, Davide Seripa, Rosanna Asselta, Donato Gemmati

**Affiliations:** 1 Department of Morphology, Surgery and Experimental Medicine and LTTA Centre, University of Ferrara, Ferrara, Italy; 2 Department of Morphology, Surgery and Experimental Medicine, University of Ferrara and Section of Internal and Cardiorespiratory Medicine, Azienda Ospedaliero-Universitaria of Ferrara, Ferrara, Italy; 3 Department of Biomedical and Specialty Surgical Sciences, and Section of Medical Biochemistry, Molecular Biology & Genetics and Ctr. Hemostasis & Thrombosis, University of Ferrara, Ferrara, Italy; 4 Department of Biomedical Sciences, Humanitas University, Rozzano (Milan) and Humanitas Clinical and Research Center, Rozzano, Milan, Italy; 5 Department of Physical Intelligence Max Planck Institute, Stuttgart, Germany; 6 Department of Medical Sciences and Ctr. Hemostasis & Thrombosis, University of Ferrara, Ferrara, Italy; 7 Operative Unit of Geriatrics, Azienda Ospedaliero-Universitaria S. Anna, Ferrara, Italy; 8 Complex Structure of Geriatrics, Research Laboratory, Department of Medical Sciences, I.R.C.C.S. Casa Sollievo della Sofferenza, San Giovanni Rotondo, Foggia, Italy; Nathan S Kline Institute, UNITED STATES

## Abstract

Cognitive impairments of different aetiology share alterations in iron and lipid homeostasis with mutual relationships. Since iron and cholesterol accumulation impact on neurodegenerative disease, the associated gene variants are appealing candidate targets for risk and disease progression assessment. In this light, we explored the role of common single nucleotide polymorphisms (SNPs) in the main iron homeostasis genes and in the main lipoprotein transporter gene (*APOE*) in a cohort of 765 patients with dementia of different origin: Alzheimer’s disease (AD) n = 276; vascular dementia (VaD), n = 255; mild cognitive impairment (MCI), n = 234; and in normal controls (n = 1086). In details, four genes of iron homeostasis (Hemochromatosis (*HFE*: C282Y, H63D), Ferroportin (*FPN1*: -8CG), Hepcidin (*HAMP*: -582AG), Transferrin (*TF*: P570S)), and the three major alleles of *APOE* (*APOE*2, *APOE*3, *APOE*4) were analyzed to explore causative interactions and synergies. In single analysis, *HFE* 282Y allele yielded a 3-fold risk reduction in the whole cohort of patients (*P*<0.0001), confirmed in AD and VaD, reaching a 5-fold risk reduction in MCI (*P =* 0.0019). The other iron SNPs slightly associated with risk reduction whereas *APOE*4 allele resulted in increased risk, reaching more than 7-fold increased risk in AD homozygotes (*P* = 0.001), confirmed to a lower extent in VaD and MCI (*P =* 0.038 and *P =* 0.013 respectively) as well as in the whole group (*P*<0.0001). Comparisons of Mini Mental State Examination (MMSE) among AD showed appreciable lowering in *APOE*4 carriers (*P =* 0.038), confirmed in the whole cohort of patients (*P =* 0.018). In interaction analysis, the *HFE* 282Y allele completely extinguished the *APOE*4 allele associated risk. Conversely, the coexistence in patients of a substantial number of iron SNPs accrued the *APOE*4 detrimental effect on MMSE. Overall, the analysis highlighted how a specific iron-allele burden, defined as different combinations of iron gene variants, might have different effects on cognitive impairment and might modulate the effects of established genetic risk factors such as *APOE*4. Our results suggest that established genetic risk factors might be affected by specific genetic backgrounds, making patients differently suited to manage iron accumulation adding new genetic insights in neurodegeneration. The recently recognized interconnections between iron and lipids, suggest that these pathways might share more than expected. We therefore extended to additional iron gene variants the newly proposed influencing mechanisms that *HFE* gene has on cholesterol metabolism. Our results have a strong translational potential promoting new pharmacogenetics studies on therapeutic target identification aimed at optimally tuning brain iron levels.

## Introduction

Alzheimer’s disease (AD), vascular dementia (VaD) and mild cognitive impairment (MCI) represent the most common forms of cognitive impairment affecting elderly people [1–3]. The comprehension of the mechanisms leading to neurodegeneration and cognitive impairment is a challenging task and inflammatory mechanisms [4–6] and iron-driven oxidative stress are key recognised physiopathological processes. Iron is essential for neuronal life but when exceeding it might result deleterious for brain cell functions [7–10] producing reactive oxygen species (ROS) and pro-inflammatory proteins [11, 12] not optimally handled in AD and MCI patients [13–15].

Increased iron levels have been reported in several neurodegenerative diseases when compared to brain of elderly healthy individuals [3, 9] and iron manipulation has been proposed as therapeutic approach for neuro-protection/restoration [16, 17]. *In vitro* and *in vivo* studies showed that one or more genetic variants or low key molecules levels might cause cell death and iron-driven oxidative stress, affecting local iron balance and healing [18–21] in different pathological settings [22, 23] rising the interest on specific single nucleotide polymorphisms (SNPs) in iron management for (neuro)degenerative diseases [24–28]. Among the genes and SNPs of iron homeostasis, only few have been widely investigated.

The High FErrum (*HFE;* HGNC:4886) hemochromatosis gene codes for a MHC class I-type protein involved in iron absorption *via* regulation of the interaction transferrin-transferrin receptor [29]. The most prevalent disease-associated gene variants in the *HFE* gene are the missense mutations C282Y and H63D, both involved in oxidative-stress and iron-dependent inflammation [23, 30]. In neurodegenerative diseases, *HFE* gene variants have been differentially linked to multiple sclerosis (MS) pathophysiology with controversial data [31–33], and we recently reported on *HFE* variants as markers of both poorer MS prognosis and higher MS aggressiveness [28]. The presence of *HFE* variants has also been associated with AD and other neurodegenerative conditions with the identification of novel connections and synergisms with the lipoprotein metabolism [23, 27, 34–36]. Though controversial results have been published so far, a recent metanalysis on AD reported no association for C282Y and protection for H63D [37].

The FerroPortiN1 (*FPN1*, also known as *SLC40A1*; HGNC:10909) gene codes for a multiple transmembrane domain protein the main cellular iron exporter in mammals [38]. *FPN1* can be regulated at many different levels including (post)-transcriptionally regulation through mRNA stability by the iron responsive element (IRE) in the 5’untranslated region (5’UTR) of mRNA [39]. *FPN1* alterations in level/function may lead to either iron deficiency or iron overload and its overexpression has been demonstrated in AD brains [40]. A recent role of FPN1 and Amyloid Precursor Protein (APP) in relation to iron/cupper homeostasis has been reported in AD supporting their combined investigation [41]. Several polymorphisms have been identified in *FPN1* gene and studied in relation to *HFE* [42] including -8CG and -98GC SNPs, close to IRE element and in complete linkage disequilibrium. *FPN1* gene variants have been investigated in pathological conditions characterized by local iron-overload [10, 27] and in different neurodegenerative diseases [28, 43].

The Hepcidin (*HAMP*, also known as Anti-Microbial Peptide; HGNC:15598) gene codes for a 25 amino acid peptide hormone that inhibits iron entry into the blood by inhibiting FPN1 protein at the cell membrane level [44]. Hepcidin is a key contributor to iron overload in cerebral ischemia [45], and an interesting hepcidin-ferroportin cooperation on iron balance has been recently demonstrated in a human in vitro neuronal model via enhanced *APP*-translation [46]. However, data about the impact of *HAMP* gene variants in neurodegenerative disease and cognitive impairments are still lacking. We recently demonstrated that a polymorphism in the *HAMP* promoter region (−582AG) plays a significant role in MS progression [28], suggesting that it might be also involved in other neurodegenerative conditions. This could be of particularly importance considering FPN1 internalization and lysosomal break down due to HAMP action with striking effects on APP-mRNA translation activity [40, 47].

The TransFerrin (*TF;* HGNC:11740) gene, codes for an iron transporter in circulating blood. The iron cellular uptake requires the binding of TF to its receptor (TFR1) at cell membrane level followed by endocytosis. While normal HFE is able to combine with TFR1 tuning its affinity for TF, HFE variants do not correctly bind the TFR, thus limiting TF-mediated iron uptake leading to both uncontrolled TF activity and increased iron uptake. Similarly, TF may result overloaded by iron influx/efflux changes due to anomalous interactions between APP and FPN1 [41, 48]. The P570S (*TF*, C1C2) genetic variant has been assessed in the context of iron balancing [49, 50] and in several neurodegenerative diseases [28, 51–53], but a synergistic effect of the *HFE* and *TF* genes also suggests possible gene-gene and gene-environment interactions [54].

Considering connections among iron metabolism and lipoprotein metabolism, we decided to investigate potential interactions between iron homeostasis SNPs and Apo-lipoproteinE (*APOE;* HGNC:613), the major recognised gene associated with AD. In particular, we included in our analysis the three most common isoforms of *APOE*: *APOE*2 (C112/C158); *APOE*3 (C112/R158); *APOE*4 (R112/R158) by allele variant discrimination. APOE is a multifunctional protein with key roles in lipid homeostasis and neurobiology [55] and it has been demonstrated that APOE2, APOE3, and APOE4 differentially stimulate APP transcription and amyloid-beta (Aβ) secretion [56]. In particular, whilst E4 is considered the most important genetic risk factor for AD, E2 is protective and E3 can be considered neutral. The pathobiological phenotype of APOE4 is characterized by age-dependent cerebral Aβ-peptide deposition, with E4 isoform promoting cerebral Aβ accumulation and the E2 involved on its clearance [57, 58]. It is of note that deregulated iron homeostasis and amyloidogenesis have been hypothesised to be synergistically connected during neurodegeneration in AD [59]. The iron dysregulation hypothesis and the amyloid cascade hypothesis coined in the context of AD, might share more than expected also in other neurodegenerative diseases and new insights from the genetic side should be taken into account [59–62].

In this work, in a cohort of elderly individuals with cognitive impairments of different origin, we evaluated the impact and role of the above described gene polymorphisms belonging to the iron and lipid homeostasis pathways, having established and mutually dependent roles in cognitive impairment pathogenesis.

## Methods

### Patient population

For patient recruitment, the study was approved by the Local Ethic Committee of “Casa Sollievo della Sofferenza”, San Giovanni Rotondo, Italy (Protocol number #3877/DS). Control samples were obtained from established anonymous collection. The Local Ethics Committee of the University-Hospital of Ferrara, Italy reviewed the use of this collection for a previous study stating that there was no need of approval since only anonymous blood samples were collected and it was impossible to identify subjects involved in the study as the link between samples and subject data was irreversibly erased (Protocol number #160519). This study has been conducted according to the Declaration of Helsinki, the Guidelines for Good Clinical Practice and the guidelines for Strengthening the Reporting of Observational Studies in Epidemiology, and it was approved by the local ethics committee for human experimentation. All patients and/or their relatives or a legal guardian in case of critically disabled demented patients were informed in detail about the research project and research protocol. Written informed consent for research was obtained from each patient, or from relatives or a legal guardian in case of critically disabled demented patients under the approval of the local ethics committee. Iron SNPs frequencies show different geographical distributions, such as in the case of the most investigated *HFE* C282Y variant that has also strong north-south and east-west gradients within the different European countries [63]. For this reason, we included only individuals with Italian ancestry that was verified/confirmed by asking the surname of parents and grandmothers. Patients consecutively attending the Complex Structure of Geriatrics, Ospedale “Casa Sollievo della Sofferenza”, San Giovanni Rotondo, Foggia were enrolled and divided in three different groups:

- AD patients (n = 276; mean age 78.46±5.7 years, males: 30.5%) as defined by the NINCDS-ADRDA criteria [64], the Global Deterioration Scale ranged from stage 3 to stage 6 and only patients with “probable” AD were included.
- VaD patients (n = 255; mean age 78.69±6.8 years; males: 35.3%) as defined by the NINDS–AIREN criteria [65], the Global Deterioration Scale ranged from stage 4 to stage 6 and only patients with “probable” VaD were included.
- MCI patients (n = 234; mean age 78.44±5.7 years, males: 43.5%) defined by the presence of short/long-term memory impairment with/without impairment in other single or multiple cognitive domains in individuals who did not meet the standardized criteria for dementia [66] and still independent in instrumental activities of daily living. Most of patients showed amnestic multi-domain MCI.

For patient recruitment, the study was approved by the Local Ethic Committee of “Casa Sollievo della Sofferenza”, San Giovanni Rotondo, Italy (protocol number # 3877/DS). As controls, we selected a total of 1086 anonymous samples age and gender matched with cases group (mean age 78.27±6.5 years, males: 36.8%) belonging to an already established anonymous collection of 3500 blood banked samples. The procedure followed the local Institutional Review Board guidelines for investigation on human biological samples. Subjects signed informed consent stating that the biological samples were collected and banked anonymously for research purposes. The Local Ethics Committee of the University-Hospital of Ferrara, Italy reviewed the use of this collection for a previous study [67] stating that there was no need of approval since only anonymous blood samples were collected and it was impossible to identify subjects involved in the study as the link between samples and subject data was irreversibly erased. The original cohort of samples did not include those with a diagnosis of established cognitive impairment, presence of severe congestive heart failure, bleeding tendency, severe liver or kidney disease, severe chronic obstructive pulmonary disease and cancer. There were no evidences of acute illnesses at the time of blood sampling.

### Clinical evaluations

At the time of enrollment personal data and clinical history were collected by trained geriatricians by a structured interview of patients and caregivers. General and neurological examinations were routinely performed. The neuropsychological assessment was performed through a battery of tests as previously described [6]. The Mini Mental State Examination (MMSE; normal score >27) was used to estimate the severity and progression of cognitive impairment and expressed as median and interquartile range [68]. All subjects underwent brain CT using a 64 volumetric scanner (GE LightSpeed VCT). The CT scan information was used to support the clinical diagnosis and to evaluate the presence of brain pathologies associated with cognitive impairment (e.g. cerebrovascular disease and normal pressure hydrocephalus). When necessary, subjects underwent brain MRI (Philips Achieva 1.5T). To exclude other causes of cognitive impairment patients were routinely monitored and clinical-chemistry analyses including serum B-12 vitamin, serum folate, liver function tests including ammonia, kidney function tests, thyroid function tests, complete blood cell count and arterial oxygen saturation were performed. Exclusion criteria: severe congestive heart failure (New York Heart Association class III-IV), severe liver or kidney disease, severe chronic obstructive pulmonary disease and cancer. There were no evidences of acute illnesses at the time of clinical observation and blood sampling.

### DNA extraction, PCR conditions, and sequencing

Venous whole blood was collected from subjects upon an overnight fast and immediately frozen at -80°C. DNA was isolated from whole blood by the automated DNA extraction and purification robot (BioRobot EZ1 system QIAGEN; Hilden, Germany), which performs purification of nucleic acids using a magnetic bead technology. *HFE*, *FPN1*, *HAMP*, *TF* and *APOE* SNPs were genotyped in the entire case-control cohorts by PCR, amplifying the relevant genomic region using specific couple of primers [28, 69]. The primers sequences together with the PCR conditions are shown in **S1 Table**, PCR were performed in a PTC-200 thermal cycler (M. J. Research, Inc., Watertown, MA, USA). SNPs detection was performed by pyrosequencing using the Pyromark ID System (Biotage AB Uppsala, Sweden) according to the standard procedures for amplicon denaturation, purification, and sequencing. The list of primers used to amplify/sequence the target genes is shown in **S1 Table**. According to our internal quality control procedures, we confirmed haplotypes by re-genotyping about 20% of randomly selected samples among each different genotype group for each specific polymorphism by means of enzymatic restriction of PCR amplicons. The restriction enzymes utilized (New England Biolabs Inc., Hitchin, UK) are listed in **S1 Table** together with the DNA fragments obtained after enzymatic digestion and the specific temperature for each different restricted amplicon. All digestion reactions were carried out according to the Supplier’s instructions. There were no discrepancies between genotypes determined in duplicate and/or by different methods. Already known genotypes were used as internal control references.

### Statistical analysis

Statistical differences among groups were assessed by Student’s t-test and Chi-squared test for mean values and genotype distribution comparisons respectively. Yates' correction or Fisher's exact test were applied when appropriate. Odds Ratio (OR) and 95% confidence interval (95% CI) were used to estimate the risk associated to different SNPs and combinations. ORs and *P*-values were calculated comparing cases *versus* controls by means of the dominant model [--/(+-/++)] and the allelic distribution [-/+]. All the remaining genetic model comparisons were also computed, though they did not show any significant differences (data not shown). All the models were considered to have a potential role on OR association and the main comparisons were further stratified by presence/absence of *APOE*4 allele in the whole group and in the different groups. Finally, the genotype distribution (--/+-/++) between cases and controls were additionally compared and *P*-value calculated. For *APOE*, presence/absence of the E4-risk-allele was the main considered discriminant in MMSE comparisons whilst all the possible genotypes were utilized for crude ORs computations. A multivariate logistic regression model was applied to calculate adjusted ORs for SNPs by means of dominant model. *P*-values ≤ 0.05 were considered significant. Deviation from Hardy-Weinberg equilibrium was calculated for each polymorphism in both cases and controls. All analyses were performed by using SPSS Statistical Package (Version 22; SPSS Inc., Chicago, IL, USA) and MedCalc statistical software (Version 17.9.7).

## Results

### Clinical and demographic characteristics of patients and controls

**Table 1** shows the main features of patients, considered as whole group or as different disease-specific subgroups, and controls. There were no differences between the whole group of patients and controls in terms of gender distribution and mean age with the exception of a very slight under-representation of males within the AD groups (*P =* 0.049). Although at different extent, the *APOE*4 allele was significantly over-represented in all case subgroups compared to controls, ascribing to AD the highest rate (*P =* 0.00001). Our cohort of patients did not show systemic iron overload or deficiency as whole group and in the different disease subgroups as well as within genders. Finally, as expected, elderly had often lower mean iron circulating levels and the MCI subgroup showed the highest median MMSE score among patients.

**Table 1:** Main clinical and demographic characteristics of patients and controls.

Variables	Whole cohort (n = 765)	AD (n = 276)	VaD (n = 255)	MCI (n = 234)	Controls (n = 1086)	*P*_W_	*P*_AD_	*P*_VaD_	*P*_MCI_
**Sex** ♂; n (%)	276 (36.0)	84 (30.5)	90 (35.3)	102 (43.5)	400 (36.8)	0.768	**0.049**	0.665	0.053
*Age	78.5±6.1	78.5±5.7	78.7±6.8	78.4±5.7	78.3±6.5	0.38	0.65	0.36	0.70
***ApoE*4** n (%)	204 (26.6)	107 (38.7)	50 (19.6)	47 (20.1)	134 (12.4)	**<0.0001**	**0.00001**	**0.0033**	**0.0032**
*Iron, μg/dL (*all ages*)	78.1±30.9	78.8±30.8	72.7±32.2	83.2±28.7	na	-	-	-	-
^#^Age<78.2	81.5±31.9	81.9±31.8	78.8±34.5	82.6±29.4	na	-	-	-	-
^#^Age≥78.2	74.6±29.4	74.4±29.3	66.4±28.5	83.8±27.9	na	-	-	-	-
**MMSE**^**§**^	21.4 (17.7–24.4)	19.7 (16.3–23.0)	19.5 (16.4–21.7)	25.3 (23.4–26.7)	na	-	-	-	-

* variables expressed as mean±SD

^**§**^ variable expressed as median and interquartile range

na: not available; *P*_w_, *P*_AD_, *P*_VaD_, and *P*_MCI_ are referred to whole cohort, AD, VaD and MCI *versus* controls respectively.

^#^ age stratified by the median value in the whole cohort. Serum iron laboratory range: 60–180 μg/dL.

### SNPs genotype distribution and crude ORs evaluation

**Table 2** shows the allele and genotype distributions of the five iron-related SNPs in the whole cohort of patients, in the three groups with different types of dementia as well in controls. Each group of cases and subgroups were statistically compared with controls for all the SNPs frequencies analyzed and *P*-values are reported. Finally, ORs were computed comparing cases *versus* controls by dominant [--/(+-/++)] and allele [-/+] distribution models.

**Table 2:** SNPs genotype distribution in patients and controls and crude ORs.

**AD (n = 276)**					
***Genotype***	***HFE* C282Y**	***HFE* H63D**	***FPN1* -8CG**	***HAMP* -582AG**	***TF* P570S**
	***n (%)***	***n (%)***	***n (%)***	***n (%)***	***n (%)***
*--*	269 (97.46)	204 (73.9)	188 (68.1)	160 (57.9)	189 (68.5)
*+-*	7 (2.5)	64 (23.2)	76 (27.5)	100 (36.2)	77 (27.9)
*++*	0 (0.0)	8 (2.89)	12 (4.34)	16 (5.79)	10 (3.6)
*P*	0.085	0.86	0.42	0.76	0.52
***Genetic model***	OR(CI;95%)	OR(CI;95%)	OR(CI;95%)	OR(CI;95%)	OR(CI;95%)
*--/(+-/++)*	0.42 (0.19–0.93) **0.031**	0.95 (0.71–1.3)	0.83 (0.62–1.09)	0.9 (0.69–1.18)	0.85 (0.64–1.12)
*-/+*	0.41 (0.18–0.91) **0.024**	0.98 (0.75–1.28)	0.86 (0.67–1.09)	0.93 (0.74–1.15)	0.87 (0.68–1.12)
**VaD (n = 255)**					
***Genotype***	***HFE* C282Y**	***HFE* H63D**	***FPN1* -8CG**	***HAMP* -582AG**	***TF* P570S**
	*n (%)*	*n (%)*	*n (%)*	*n (%)*	*n (%)*
*--*	249 (97.6)	180 (70.6)	188 (68.1)	160 (62.7)	167 (65.49)
*+-*	6 (2.35)	65 (25.5)	60 (21.7)	81 (31.7)	74 (29.0)
*++*	0 (0.0)	10 (3.9)	7 (2.5)	14 (5.49)	14 (5.49)
*P*	0.077	0.40	**0.0096**	0.099	0.43
***Genetic model***	OR(CI;95%)	OR(CI;95%)	OR(CI;95%)	OR(CI;95%)	OR(CI;95%)
*--/(+-/++)*	0.39 (0.16–0.91) **0.026**	1.13 (0.84–1.53)	0.63 (0.46–0.85) **0.0033**	0.74 (0.56–0.98) **0.04**	0.97 (0.73–1.29)
*-/+*	0.38 (0.16–0.89) **0.020**	1.16 (0.89–1.5)	0.66 (5.5–0.86) **0.0018**	0.8 (0.63–1.01) 0.07	1.03 (0.9–1.31)
**MCI (n = 234)**					
***Genotype***	***HFE* C282Y**	***HFE* H63D**	***FPN1* -8CG**	***HAMP* -582AG**	***TF* P570S**
	*n (%)*	*n (%)*	*n (%)*	*n (%)*	*n (%)*
*--*	231 (98.7)	172 (73.5)	147 (62.8)	145 (61.9)	163 (69.6)
*+-*	3 (1.28)	59 (25.2)	72 (30.7)	79 (33.7)	62 (26.49)
*++*	0 (0.0)	3 (1.3)	15 (6.4)	10 (4.3)	9 (3.84)
*P*	**0.016**	0.52	0.66	0.17	0.34
***Genetic model***	OR(CI;95%)	OR(CI;95%)	OR(CI;95%)	OR(CI;95%)	OR(CI;95%)
*--/(+-/++)*	0.21 (0.06–0.67) **0.0024**	0.98 (0.71–1.35)	1.05 (0.78–1.4)	0.76 (0.57–1.02)	0.80 (0.59–1.08)
*-/+*	0.2 (0.06–0.66) **0.0019**	0.94 (0.7–1.25)	1.08 (0.84–1.37)	0.79 (0.62–1.01) 0.066	0.85 (0.65–1.1)
**Whole cohort of cases (n = 765)**					
***Genotype***	***HFE* C282Y**	***HFE* H63D**	***FPN1* -8CG**	***HAMP* -582AG**	***TF* P570S**
	*n (%)*	*n (%)*	*n (%)*	*n (%)*	*n (%)*
*--*	749 (97.8)	556 (72.67)	523 (68.3)	465 (60.8)	519 (67.8)
*+-*	16 (2.22)	188 (24.57)	208 (27.18)	260 (34.0)	213 (27.8)
*++*	0 (0.0)	21 (2.74)	34 (4.44)	40 (5.2)	33 (4.31)
*P*	**<0.0001**	0.93	0.13	0.078	0.266
***Genetic model***	OR(CI;95%)	OR(CI;95%)	OR(CI;95%)	OR(CI;95%)	OR(CI;95%)
*--/(+-/++)*	0.34 (0.13–0.6) **<0.0001**	1.01 (0.83–1.26)	0.82 (0.67–0.99) **0.047**	0.8 (0.67–0.97) **0.025**	0.87 (0.7–1.05)
*-/+*	0.34 (0.19–0.59) **<0.0001**	1.03 (0.86–1.23)	0.85 (0.72–1.0) 0.06	0.84 (0.72–0.98) **0.034**	0.91 (0.77–1.08)
**Controls (n = 1086)**					
***Genotype***	***HFE* C282Y**	***HFE* H63D**	***FPN1* -8CG**	***HAMP* -582AG**	***TF* P570S**
	*n (%)*	*n (%)*	*n (%)*	*n (%)*	*n (%)*
*--*	1023 (94.2)	794 (73.1)	694 (63.9)	603 (55.5)	704 (64.8)
*+-*	61 (5.6)	265 (24.4)	338 (31.1)	418 (38.5)	340 (31.3)
*++*	2 (0.2)	27 (2.5)	54 (5)	65 (6)	42 (3.9)

-- and ++ indicate homozygotes for the common and polymorphic (rare) allele respectively; + - indicates heterozygotes; dominant [--/(+-/++)] and allele [-/+] genetic models were used for ORs computation as described in the Methods section; significant *P*≤0.05 are shown in bold.

As for *HFE* C282Y, the 282Y-allele yielded a 3-fold risk reduction in the whole cohort of patients (OR = 0.34; 0.19–0.59; *P*<0.0001). The protective effect was confirmed at the same extent in the AD and VaD subgroups and almost reaching a 5-fold risk reduction among the MCI subgroup (OR = 0.2; 0.06–0.66; *P =* 0.0019). Among the other iron SNPs investigated, a significant risk reduction was also observed in the VaD subgroup either for *FPN1* -8G-carriers (OR = 0.63; 0.46–0.85; *P =* 0.0033) and *HAMP* -582G-carriers (OR = 0.74; 0.56–0.98; *P =* 0.04) computing together homozygous and heterozygous cases. Both associations showed a similar strength in the whole cohort of patients (OR = 0.82; 0.67–0.99; *P =* 0.047 and OR = 0.8; 0.67–0.97; *P =* 0.025 respectively).

The presence of the *APOE*4 allele gave significant ORs in all the subgroups and in the whole cohort when performing an indiscriminate comparison of any *APOE*4 allele condition *versus* the remaining genotypes. However, in consideration of the recognised protective effect of *APOE*2 against AD and other neurodegenerative diseases, we calculated in detail *APOE*-allele specific ORs considering the E3/E3 as reference class (**S2 Table**). As expected, those AD patients homozygous for the *APOE*4 allele showed the highest risk association (OR = 7.56; 2.27–24.15; *P =* 0.001). Although at different extent, appreciable increasing risks were also observed in the VaD and MCI subgroups and in the whole cohort of cases.

Since ORs did not change considering or not the E2/E4 genotype from the global E4(+) computation (OR: 2.79; 2.18–3.57 *versus* 2.58; 2.02–3.28, respectively), we left the E2/E4 genotype in all subsequent analyses.

All the genotypes of the polymorphisms investigated were distributed according to the Hardy-Weinberg equilibrium.

### Multivariate logistic regression analyses

To support the above described results, and to exactly weight the role of each iron variable considered, we performed a multivariate logistic regression model taking into account the variables associated to dementia. As shown in **Table 3**, *HFE* C282Y gave the strongest association in each subgroup considered confirming the crude ORs showed in **Table 2**, though AD yielded a borderline value. *FPN1* -8CG confirmed its highest association found in the VaD subgroup as well as *HAMP* -582AG did in VaD and in the whole cohort. The remaining SNPs did not reach significant associations.

**Table 3:** Multivariate logistic regression analysis.

Iron SNPs	Whole cohort (n = 765)	AD (n = 276)	VaD (n = 255)	MCI (n = 234)
	*OR(CI;95%); P*	*OR(CI;95%); P*	*OR(CI;95%); P*	*OR(CI;95%); P*
***HFE* C282Y**	0.37(0.21–0.64); **0.0005**	0.48(0.22–1.04); 0.06	0.38(0.16–0.91); **0.029**	0.21(0.06–0.69); **0.010**
***HFE* H63D**	1.01(0.82–1.25): 0.86	0.92(0.67–1.26); 0.61	1.11(0.82–1.51); 0.470	0.96(0.69–1.32); 0.811
***FPN1* -8CG**	0.82(0.67–1.00); **0.050**	0.83(0.62–1.12); 0.22	0.62(0.46–0.85); **0.0028**	1.05(0.78–1.41); 0.724
***HAMP* -582AG**	0.79(0.66–0.96); **0.022**	0.88(0.66–1.17); 0.33	0.73(0.55–0.96); **0.0029**	0.77(0.58–1.04); 0.08
***TF* P570S**	0.85(0.69–1.03); 0.111	0.79(0.59–1.06); 0.127	0.96(0.71–1.28); 0.781	0.81(0.59–1.10); 0.188

ORs values have been adjusted taking into account sex and *APOE*4-allele distribution.

### MMSE score in patients stratified by type of dementia and SNPs genotype

MMSE comparisons, stratified by the different iron SNPs genotypes, did not reveal any significant result neither within the different subgroups of patients or within the whole group of cases. Slight differences that deserve to be further investigated are those observed among AD and VaD subgroups for *FPN1* -8CG (*P =* 0.062 and *P =* 0.08 respectively), for *HFE* C282Y and H63D (*P =* 0.074 and *P =* 0.099 respectively). Similar MMSE analysis, stratified by presence/absence of *APOE*4-allele, yielded different MMSE score just in the whole group and in AD subgroup. Accordingly, AD patients carrying the *APOE*4-allele showed significant lower MMSE median and interquartile range values compared with those carrying the remaining *APOE*-alleles (18.7, 15.8–21.3 *versus* 20.4, 16.5–23.4; *P =* 0.038). Even though at a lower extent, a similar trend was observed in the whole group of patients (*P =* 0.018) **(S3 Table)**.

### Effect on ORs evaluation of iron gene SNPs-APOE4 allele (coupled analysis)

The observation that *HFE* C282Y SNP and *APOE*4-allele were the only ones strongly associated in all the subgroups of patients respectively with risk reduction and risk increasing, prompted us to perform a risk assessment by coupling each polymorphic allele in the iron genes with the *APOE*4 allele to disclose potential mutual influences. As shown in **Table 4**, no additive or multiplicative effects were observed; on the contrary the coexistence of the *APOE*4 allele with any other among the iron SNPs led to a decreased risk with respect to the *APOE*4 risk obtained in single analysis (**S2 Table**). The effect was maximum among those co-carrying the *HFE* 282Y allele, which completely extinguished the *APOE*4 associated risk, while a partial but still appreciable effect was observed with the remaining iron SNPs according to their strength showed in the single analysis (**Table 2**).

**Table 4:** ORs evaluation in patients carrying both *APOE*4 allele and any other polymorphic allele among the iron SNPs.

Cognitive diagnosis		ALLELES COMBINATIONS*									
		*HFE*/*APOE*		*HFE*/*APOE*		*FPN1*/*APOE*		*HAMP*/*APOE*		*TF*/*APOE*	
		(282Y/E4)		(63D/E4)		(-8G/E4)		(-582G/E4)		(570S/E4)	
		OR	*P*	OR	*P*	OR	*P*	OR	*P*	OR	*P*
		(CI;95%)		(CI;95%)		(CI;95%)		(CI;95%)		(CI;95%)	
**AD**	*(a)*	0.98	n.s.	2.36	**0.0018**	2.52	**0.0003**	3.05	**0.0001**	3.75	**0.0001**
		(0.21–4.6)		(1.41–3.93)		(1.57–4.06)		(2.01–4.61)		(2.40–5.85)	
	*(b)*	1.37	n.s.	3.27	**0.0001**	2.39	**0.0009**	2.95	**0.0001**	4.3	**0.0001**
		(0.3–6.5)		(1.93–5.55)		(1.47–3.89)		(1.89–4.62)		(2.71–6.85)	
**VaD**	*(a)*	0.53	n.s.	1.27	n.s.	1.29	n.s.	1.48	n.s.	1.98	**0.016**
		(0.06–4.2)		(0.7–2.39)		(0.72–2.34)		(0.86–2.48)		(1.16–3.38)	
	*(b)*	0.56	n.s.	1.45	n.s.	1.65	0.11	1.06	n.s.	1.99	**0.015**
		(0.07–4.48)		(0.76–2.77)		(0.90–3.03)		(0.62–1.80)		(1.16–3.45)	
**MCI**	*(a)*	n.a.		1.17	n.s.	1.52	n.s.	1.21	n.s.	1.51	n.s.
				(0.6–2.29)		(0.85–2.72)		(0.69–2.14)		(0.84–2.76)	
	*(b)*	n.a.		1.29	n.s.	1.68	0.086	0.93	n.s.	1.50	n.s.
				(0.65–2.57)		(0.93–3.06)		(0.51–1.67)		(0.82–2.77)	
**Whole** **cohort**	*(a)*	0.53	n.s.	1.62	**0.0023**	1.79	**0.0038**	1.92	**0.0002**	2.43	**0.0001**
		(0.14–2.0)		(1.07–2.46)		(1.22–2.64)		(1.36–2.72)		(1.67–3.55)	
	*(b)*	0.61	n.s.	1.95	**0.0025**	1.95	**0.001**	1.54	**0.02**	2.55	**0.0001**
		(0.16–2.32)		(1.27–2.99)		(1.32–2.91)		(1.08–2.22)		(1.73–3.75)	

*, double carriers for each couple of SNPs considered (rare alleles) were compared with the rest of genotypes *(a)* and with homozygotes for both the common alleles in the couple of SNPs considered *(b)*.

n.a., not applicable due to the absence of MCI patients carrying both *APOE*4 and *HFE C282Y*. n.s., not statistically significant.

### Combined analysis of iron SNPs and ORs evaluation stratified by APOE4 condition

To investigate possible cumulative effects or mutual interactions among iron SNPs, we calculated the risk resulting from the coexistence in patients of at least three polymorphic allele in the iron genes (multicarriers) stratified by the presence/absence of the *APOE*4 allele (**S4 Table, panel A**). The OR-values were firstly calculated comparing the whole group of cases and controls regardless to the *APOE* condition (OR1), this analysis did not show any significant protection towards cognitive impairment. Accordingly, when the risk was calculated considering only those cases and controls who did not carry the *APOE*4 (OR2), a weak risk protection was obtained in AD patients (OR = 0.65; 0.43–0.97; *P =* 0.035) and in the whole group of cases (OR = 0.74; 0.57–0.97; *P =* 0.030), whilst no significant risk reduction was observed in VaD and MCI.

Since coexistence of multiple iron SNPs in our cohort of patients provided evidence of a subtle protective role, we investigated if the opposite gene condition *(i*.*e*. no polymorphic alleles in iron genes) was associated with significant risk variations and, as done before, we further stratified by the presence/absence of the *APOE*4 allele. As shown in **S4 Table, panel B**, the OR-values were firstly calculated comparing the whole groups of cases and controls regardless to the *APOE* condition (OR1) showing significant risk association in all patient groups with the exception of MCI. By considering only those cases and controls positive for *APOE*4 allele, a massive increase in the risk was observed (OR3), reaching more than five-fold risk association among AD patients (OR = 5.11; 2.74–5.52; *P*<0.0001) which was higher than that obtained in the single analysis (OR = 4.49; 3.32–6.08; **S2 Table**). A similar improvement was observed in the rest of patients and in the whole group. Then the existence of an individual genetic background characterized by presence or absence of a substantial polymorphic iron-allele burden might differently (counter)act on other inherited established risk factors.

### MMSE comparison according to *iron-allele burden* stratified by *APOE*4 condition

To explore whether *APOE*4 allele might differentially influence the MMSE score when surrounded by “opposite” genetic iron settings, we stratified patients according to the number of polymorphic iron genes (*i*.*e*. multicarriers *versus* no polymorphic alleles) and further by the presence/absence of *APOE*4 allele (**S5 Table**).

The number of patients carrying opposite genetic iron setting was equally distributed among the whole group of cases (OR = 1.05, 0.81–1.36) and in the different subgroups (AD: OR = 1.0, 0.65–1.51; VaD: OR = 1.11, 0.71–1.73; MCI: 1.06, 0.66–1.72). Interestingly, the median MMSE score did not significantly differ among the groups of patients stratified according to opposite iron-allele burden (**S5 Table, panel A**: multiple iron SNPs *versus* **S5 Table, panel B**: no iron SNPs), neither in the subgroups nor in the whole cohort. However, in the AD group those carrying multiple iron SNPs showed a lower median value than their counterpart negative for the iron SNPs (MMSE: 18.7; 15.3–22.5 *versus* 20.2; 17.6–22.9; *P =* 0.098) and this variation was more evident within the *APOE*4(+) subgroups of AD (MMSE: 17.4; 15.4–19.9 *versus* 19.0; 17.6–22.9; *P* = 0.038). Of interest, although a substantial polymorphic iron-allele burden seems do not affect the MMSE in the whole group (MMSE: 21.0, 17.4–24.4 *versus* 21.4, 18.5–23.7; *P =* 0.27) as well as in subgroups (AD: *P =* 0.098; VaD: *P =* 0.44; MCI: *P =* 0.45), the *APOE*4 significantly worsened the median MMSE of the whole group only in the presence of an elevated iron allele-burden (*P =* 0.006) with respect to the opposite genetic counterpart (*P =* 0.38).

### Peripheral iron levels stratification by iron SNPs according to different genotypes

Although the poor relationship between dementia and circulating iron level, this is the most commonly assessed in the clinical routine among the iron parameters. As shown before, our cohort of patients did not have systemic iron overload or deficiency with MCI showing the highest mean values (MCI *versus* AD: *P =* 0.09; MCI *versus* VaD: *P =* 0.026). Normally, males have higher iron levels compared to females, accordingly in our dementia patients this was confirmed in VaD (76.80±35.89 μg/dL and 70.42 ±29.85 μg/dL in male and female respectively; *P =* 0.065) and in MCI subgroup (87.00±30.81 μg/dL and 80.23±26.64 μg/dL in male and female respectively; *P =* 0.037). Conversely, the two genders belonging to the AD subgroup showed completely overlapped iron levels (78.73±29.11 μg/dL and 78.78±31.56 μg/dL in males and females respectively; *P =* 0.495). Peripheral iron dyshomeostasis does not necessarily cause neurodegeneration or reflect brain iron deposit causing no genetic connection with neurodegeneration [70], and the common iron SNPs usually investigated are not considered strong determinants of iron accumulation [71, 72]. On the contrary, *APOE*4-allele has been widely confirmed as key determinant of brain iron deposit in dementia also contributing to cognitive decline [73–77]. For this purpose, we stratified peripheral iron levels by SNPs and none of the SNPs in the iron hemostasis or APOE genes yielded significant differences in mean levels (**Table 5**). In an explorative way, we also investigated the presence of different genotype distributions between sexes within the different cognitive disease. No gender-related differences in genotype distributions were found in the whole group and in the different diseases subgroups (data not shown).

**Table 5:** Peripheral iron levels stratified by SNPs genotypes.

*Genotype*	*HFE* C282Y	*HFE* H63D	*FPN1* -8CG	*HAMP* -582AG	*TF* P570S	*APOE*4
	μg/dL (mean±SD)	μg/dL (mean±SD)	μg/dL (mean±SD)	μg/dL (mean±SD)	μg/dL (mean±SD)	μg/dL (mean±SD)
*--*	78.1±30.2	77.4±30.2	77.6±31.9	77.7±31.5	77.6±31.3	77.1±29.8
*+-/++*	78.2±27.9	79.9±31.7	78.2±21.2	77.2±29.9	79.2±30.1	80.7±33.5
*P*	0.98	0.30	0.54	0.50	0.49	0.16

## Discussion

The iron hypothesis proposes Aβ pathogenicity *via* interaction with specific metals mainly unbalanced by suboptimal hepcidin-ferroportin axis [62, 78–81] while the amyloid cascade hypothesis considers amyloidosis due to an abnormal Aβ deposit/clearance as the primary pathological event [11, 60, 61]. Amyloidosis, has been recently demonstrated differently tuned by the different *APOE*-alleles [79] in AD and in cognitively normal older adults [82] and the two hypotheses might share more than expected also in other neurodegenerative diseases with mutual causative interactions.

We found that the presence of the rare *HFE* 282Y-allele had a significant 3-fold risk reduction in all groups almost reaching a 5-fold reduction in MCI. These results are in accordance with previous reports ascribing to 282Y-allele a protective role against neurodegenerative disease [83, 84], though the recent metanalysis of Lin et al. did not confirm such a protection [37]. The *HFE* H63D polymorphism did not show any significant risk fluctuation in our study, though it has been reported a higher rate of the 63D-allele in neurodegenerative disease [85], also in combination with different *APOE* isoforms [86–88], suggesting H63D a genetic risk factor for neurodegenerative diseases [89]. Conversely, the same metanalysis of Lin et al. supported a slight protective role of H63D [37], underlying the still conflicting results.

Brain iron load increases with age leading to oxidative stress that could progress towards neurodegeneration and cognitive decline in an unfavourable genetic *milieu*. Among younger individuals, brain iron levels sharply increase with low interindividual variance. Conversely, in midlife, brain iron concentration reaches a plateau showing higher interindividual variance [11]. In this scenario, gene-gene and gene-environment interactions could play a key role in the progression to cognitive impairment. Therefore, additional SNPs among key genes controlling iron homeostasis might be involved, as demonstrated by our group in a study focused on MS patients [28]. To gain insights on iron-dependent processes in cognitive impairment, we extended the analyses to further SNPs: *FPN1* -8CG, *HAMP* -582AG and *TF* P570S.

We found that *FPN1* -8G-allele was underrepresented in VaD patients, yielding a significant 1.5-fold risk reduction (*P*<0.0018), while a borderline reduction was observed among the whole cohort of patients (*P =* 0.047). In the same fashion, *HAMP* -582AG SNP was underrepresented among VaD subgroup (*P =* 0.04) as well in the whole cohort of cases (*P =* 0.025; computing together -582AG heterozygotes and -582GG homozygotes). Controversial results exist on the association between *TF* P570S and AD [51, 52], and a suggestive synergism has been reported between *TF* and *HFE* gene variants in AD patients [54]. In addition, a more recent study investigating genetic and biochemical markers in patients with AD associated several intronic variants in *FPN1*, *TF* and *TF*R genes, concluding that iron accumulation and oxidative damage might contribute to AD pathophysiology [43]. That study also found low circulating iron levels in the same subset of patients, suggesting a new iron-mediated mechanism in neurodegeneration. The reduced rate of iron excretion from cells could explain the intracellular iron overload and at the same time the increased production of toxic ROS together with the low circulating iron levels. This suggests that peripheral iron assessment could be a novel and effective early biomarker for treatment monitoring [43], although in our study we did not find any association.

How iron SNPs responsible for cellular iron retention could protect against cognitive diseases is not easy to clarify. Different brain regions could differently suffer from changes in iron homeostasis because of unequal “equipment” in iron receptors/transporters. Interestingly, iron surplus would enhance cell viability making cells more resistant to other toxic metals, through improved *APP* and ferritin translation, as demonstrated *in vitro* on Pb-induced damage and *in vivo* against Pb-toxicity [46].

Among the major genetic risk factors for AD, there is the *APOE* in the form of the *APOE*4 allele. It was not only involved in the amyloid cascade promotion but it has also been recently investigated as responsible for ROS production in AD and MCI patients [80] as well as for worsening memory performance in relation to Aβ-levels in cognitively normal adults [82].

Accordingly, we firstly investigated the risk-association for the presence of the *APOE*4 allele. As expected, in AD the associated risk was higher, reaching a quite 4.5-fold rising (*P*<0.0001), whilst among MCI and VaD the risk was less than 1.8-fold higher (*P =* 0.0003). Finally, *APOE4* homozygotes in AD had risk values higher than 7.5-fold (*P* = 0.001). When we looked at the MMSE comparison stratifying patients according to the presence/absence of the *APOE*4 allele, the median MMSE was significantly lower (*P =* 0.038) only in AD patients who carried the *APOE*4 compared to non-carriers. A slight difference was also found in the whole cohort of cognitive impaired patients (*P =* 0.018).

Despite the unquestionable key role that iron genes have on neurodegenerative disease pathogenesis, iron SNPs have not been widely investigated, with the only exception of *HFE* and TF polymorphisms that are reported in dedicated meta-analyses (**S6 Table**). We hence explored if the coexistence of more than one gene variants could disclose possible cumulative interactions with significant effects on the susceptibility risk and on MMSE score. We firstly coupled each polymorphic allele in the iron genes with the *APOE*4 allele and recomputed the risk association. Given that the rate of the combined existence of any polymorphic iron allele with the *APOE*4 radically dropped off in patients and that the new combined risk association was inversely related to the strength of the iron allele, we can exclude that iron SNPs and *APOE*4 could synergize, as differently reported by other authors [86–88]. On the contrary, we could speculate that a protective effect of iron SNPs might potentially counteract the *APOE*4 risk feature. As expected, the *HFE* 282Y allele, showing the highest protective effect within our patients, had the maximum effect on the coupled risk computation if compared to *TF* 570S allele that virtually had no consequence in similar risk calculation. As an example, the crude OR scored for the *APOE*4 allele in AD patients (OR_(APOE4)_: 4.49; 3.32–6.08) was unmodified by the co-presence of the *TF* 570S allele (OR_(APOE4+*TF*570S)_: 3.75; 2.40–5.85), while it completely extinguished when *HFE* 282Y allele was coinherited (OR_(APOE4+*HFE*282Y)_: 0.98; 0.21–4.6). Also considering the extreme rare frequency of the combined coinheritance of the double-carrier status (i.e. *HFE* 282Y/*APOE*4), we could speculate an effect on the *APOE*4 allele risk association as follows: *HFE* C282Y>*HFE* H63D>*HAMP* -582AG>*FPN1* -8CG>*TF* P570S.

Considering that iron-SNPs seem to account for risk reduction, we combined them together in a collective risk evaluation analysis. We firstly attempted at evaluating any possible interaction among the iron SNPs, providing that subjects carried at least three polymorphic alleles among the four iron genes. We then calculated the associated risk and the median MMSE scores. Concerning the combined risk association, no appreciable variations were recorded, though the exclusion of the APOE4 allele from the computation yielded a significant risk reduction in AD patients and in the whole group (*P =* 0.035 and *P =* 0.03 respectively). We therefore explored the “other side of the coin” and considered the absence of any polymorphic allele in the iron genes as a risk condition. This particular status yielded risk association ranging from 1.3 to more than 1.5-fold in all the subgroups, and unexpectedly the risk for *APOE*4 allele improved, reaching more than 5-fold in AD. As for the MMSE median scores, the *APOE*4 allele completely lost its detrimental effect on MMSE in the absence of a substantial polymorphic iron-allele burden. In fact, though the median total MMSE did not differ among the opposite iron-SNPs conditions (MMSE_(3-iron SNPs)_: 21.0; 17.4–24.5 *versus* MMSE_(No-iron SNPs)_ 21.4; 18.5–23.7; *P =* 0.27), the inheritance of the *APOE*4 allele had significant detrimental consequences on the MMSE score of the whole group only among those who co-carried a substantial iron allele burden (*P =* 0.006).

**Fig 1:**
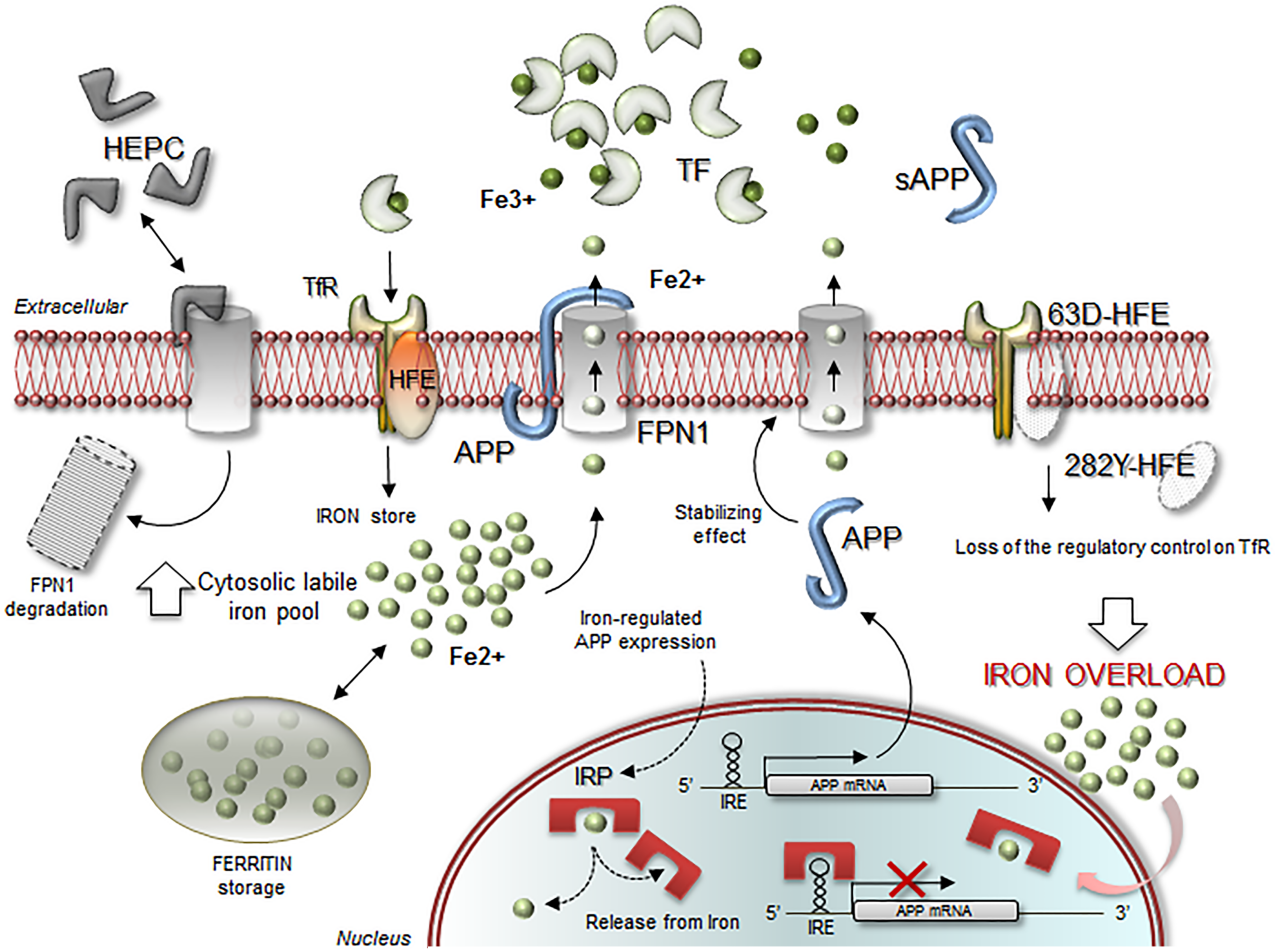
Schematic model linking HFE, HAMP and TF to FPN1-APP complex in detoxifying action for balanced iron homeostasis. The picture highlights the main pathways and molecular mediators involved in iron homeostasis maintenance by FPN1-APP cooperation. HAMP is Hepcidin; TF is Transferrin; TF-R is Transferrin receptor; HFE is Hemochromatosis; FPN is Ferroportin; IRP and IRE are Iron Regulatory Protein and Iron Responsive Element respectively.

Overall, our results ascribe to iron genes variants a significant role in cognitive disease assigning from one hand a protective effect and on the other providing a suitable environment to maximize the APOE4 detrimental effects on cognitive capacity, being amplified in patients carrying a high iron-allele burden. Obviously, these observations deserve further confirmation in a larger cohort of patients. The poor strength of the results due to sub-sampling in the stratified analyses must be considered, even though the whole cohort completely resumes the single observations within the subgroups. Anyway, a possible interpretation of these opposite findings may be in part explained by the recently proposed role of *APOE* as allele-specific antioxidant molecule, with *APOE*4 less efficient in binding iron and reducing oxidative stress than *APOE*3, particularly when surrounded by a substantial toxic metal burden [73, 74]. Local iron overload goes together with increased ROS, and *HFE* variants fail in counteracting cellular iron uptake as confirmed by several biological/instrumental assessments correlating iron overload, severity of cognitive impairment and *HFE* gene condition [90–92]. How iron-accumulating variants in *HFE* can protect against AD or other neurodegenerative disease still remains unclear. One possible explanation could rise from the observation that in AD iron is most dense in proximity of Aβ plaques [93], and promotes Aβ deposition [94]. Although the neurotoxicity of Aβ is enhanced by iron it also depends on the iron export action of APP itself [41]. Recently discovered, APP mRNA contains a functional 5’UTR-IRE, similar to that found in *FPN1* and *TF*. Consequently, intracellular iron availability can increase *APP*, *FPN1* and *TF* translation and, at high levels iron facilitates the cooperative APP-FPN1 action improving iron efflux (**Fig 1**), hampering the detrimental APP maturation and Aβ deposition [41]. This fine equilibrium is a recent therapeutic target for AD, aimed at generating optimal intracellular iron levels and optimizing brain iron homeostasis to contrast Aβ-genesis [41, 48, 95, 96]. APP upregulation, relevant for AD, is a key element also in general brain iron homeostasis since it actively proceeds during vascular accidents protecting in turn neurons from heme release in ischemic/haemorrhagic stroke, ascribing it a great value also for VaD patients [97, 98]. Another neurodegenerative disease in which iron SNPs play a crucial role is MS [28, 30–33, 99]. In fact, although MS is a myelin-degeneration disease characterized by inappropriate immune responses, a strong relationship between iron and myelin exists [100]. Iron related genes and myelin related genes share indeed iron dysregulation as common pathogenic pathway. Myelin degeneration can occur early as consequence of brain iron dyshomeostasis. Subsequently, the lack of adequate counteracting mechanisms, in part genetically determined, may cause exacerbation/progression of the disease via oxidative stress mechanisms [28, 99]. In line with this, APP is crucial also for MS being upregulated in damaged axons and emerging as a promising therapeutic target [101]. Finally, a recent study evaluating results summarized from GWAS meta-analyses on circulating iron levels and common variants affecting iron homeostasis on AD risk did not identify any genetic relationship ascribing to peripheral iron a no causal role in initiation of AD, rather suggesting ferritin as leader mechanism by which *APOE*4 is considered a risk factor for AD [70].

In conclusion, although this study requires confirmation in larger cohorts of patients and in longitudinal analyses, our results strongly suggest that established genetic risk factors might be modulated by specific genetic backgrounds, making patients differently suited to manage iron overload thus resulting in different clinical phenotypes. In line with the recently recognized interconnections between iron and lipids, we have here extended to other iron gene variants the newly proposed influencing role of *HFE* gene on cholesterol metabolism with a strong translational potential, supporting pharmacogenetics studies aimed at optimally tuning intracellular iron levels to optimize brain iron homeostasis.

## Supporting information

S1 Table Primer sequences, restriction-product characteristics and PCR conditions. (DOCX)

S2 Table Crude ORs evaluation in patients stratified by APOE allele combinations. (DOCX)

S3 Table Mini mental state examination (MMSE) in patients stratified by type of dementia and SNPs genotypes. (DOCX)

S4 Table ORs evaluation comparing multicarriers (A) or no carriers (B) of polymorphic alleles in iron genes stratified by *APOE*4 condition.
(DOCX)

S5 Table MMSE comparison in patients multicarrier (A) or no carriers (B) of polymorphic alleles in iron genes stratified by *APOE*4 condition.
(DOCX)

S6 Table Summary ORs from selected meta-analyses on AD and HFE and TF SNPs. (DOCX)
