# Supplementary material for: Gene-gene interactions among coding genes of iron-homeostasis proteins and APOE-alleles in cognitive impairment diseases
Source: PLoS One. 2018 Mar 8;13(3):e0193867. doi: 10.1371/journal.pone.0193867 (PMC5843269; doi:10.1371/journal.pone.0193867)
Supplement: S1 Table — (DOCX) [file pone.0193867.s001.docx]

**S1 Table. Primer sequences, restriction-product characteristics and PCR conditions.**

| **Oligo name** | **Oligo sequence** | **PCR size (bp)** | **Restriction enzyme** | **Restriction**  **products (bp)** | **PCR thermal profiles** |
| --- | --- | --- | --- | --- | --- |
| ***HFE* C282Y**  Fw Rst  Rv Rst  Fw Py  Rv Py  Sq | (rs 1800562)  5’-TGGCAAGGGTAAACAGATCC-3’  5’-CTCAGGCACTCCTCTCAACC-3’  5’-CGAACCTAAAGACGTATTGCC-3’  5’-CCCAATAGATTTTCTCAGCTCCT-3’[Bio]  5’GGAAGAGCAGAGATATACG-3’ | 387 | *Rsa*I  (37°C) | 247+140 (*WT*) | 95°C/2', 95°C/30", 53.4°C/40", 72°C/40", 72°C/5' (34 cycles) |
| ***HFE* H63D**  Fw Rst  Rv Rst  Fw Py  Rv Py  Sq | (rs 1799945)  5’-ACATGGTTAAGGCCTGTTGC-3’  5’-GCCACATCTGGCTTGAAATT-3’  5’-CCACATCTGGCTTGAAATTCT-3’  5’-GTTTGAAGCTTTGGGCTACG-3’[Bio]  5’GGGCTCCACACGGCG-3’ | 207 | *Bcl*I  (50°C) | 137+70 (*P*) | 95°C/2', 95°C/30", 51.6°C/30", 72°C/45", 72°C/5' (34 cycles) |
| ***FNP1* -8CG**  Fw Rst/Py  Rv Rst/Py  Sq | (rs 11568351)  5’CCAGTTCCTTGCACTCCTG-3’  5’CATCCTCTCTGGCGGTTG-3’[Bio]  5’AGAGCCAGCGGGGTC-3’ | 129 | *BstU*I  (60°C) | 85+44 (*P*) | 95°C/2', 95°C/30", 52°C/40", 72°C/40", 72°C/5' (34 cycles) |
| ***HAMP* -582AG**  Fw Rst  Rv Rst  Fw Py  Rv Py  Sq | (rs 10421768)  5’-ACCCTCCTGCCTTGGCCTC-3’  5’-CCATTGCTTTAAGCTCTCACC-3’  5’-ACATCTCAAGGGTCTGACACTGG-3’  5’-GAGCAGGGCAAGCATCAGC-3’[Bio]  5’-TCTGACACTGGGAAAAC-3’ | 252 | *HpyCH4*IV  (37°C) | 226+26 (*P*) | 95°C/2', 95°C/30", 54.3°C/30", 72°C/20", 72°C/5' (34 cycles) |
| ***TF* P570S**  Fw Rst  Rv Rst  Fw Py  Rv Py  Sq | (rs 1049296)  5’-GCTGTGCCTTGATGGTACCAGGTAA-3’  5’-GGACGCAAGCTTCCTTATCT-3’  5’-GAAAAAGACTATGAGTTGCTGTGC-3’  5’-CTGTGACCACAGCGTGATTC-3’[Bio]  5’-TGATGGTACCAGGAA-3’ | 110 | *BstE*II  (60°C) | 89+21 (*WT*) |  |
| ***APOE***  Fw Rst  Rv Rst  Fw Py  Rv Py  Sq C112R  Sq R158C | (C112R: rs 429358; R158C: rs 7412)  5’-ACAGAATTCGCCCCGGCCTGGTACAC-3'  5'-TAAGCTTGGCACGGCTGTCCAAGGA-3'  5’-CTGGGCGCGGACATGGAG-3’  5’-CCCCGGCCTGGTACACTG-3’[Bio]  5’-CGGACATGGAGGACG-3’  5’-CCGATGACCTGCAGA-3’ | 244 | *HhA-I* | *multiple size products** | 95°C/2', 95°C/30", 57°C/15", 72°C/15", 72°C/5' (34 cycles) |

Fw and Rv, indicate the forward and reverse primer respectively; Sq, indicates the specific sequencing primer; Rst and Py, indicate restriction and Pyrosequencing technique respectively; *WT* and *P,* indicate the wild-type (common) and polymorphic (rare) allele respectively; [Bio], indicate the biotinylated primer; * according to [28, 69].
