# Supplementary material for: Gene-gene interactions among coding genes of iron-homeostasis proteins and APOE-alleles in cognitive impairment diseases
Source: PLoS One. 2018 Mar 8;13(3):e0193867. doi: 10.1371/journal.pone.0193867 (PMC5843269; doi:10.1371/journal.pone.0193867)
Supplement: S2 Table — (DOCX) [file pone.0193867.s002.docx]

**S2 Table. Crude ORs evaluation in patients stratified by *APOE* allele combinations.**

| **APOE** | | **Cognitive diseases** | | | | | | | | | | | | **Controls** (n=1086) |
| --- | --- | --- | --- | --- | --- | --- | --- | --- | --- | --- | --- | --- | --- | --- |
|  |  | **AD** (n=276) | | | **VaD** (n=255) | | | **MCI** (n=234) | | | **Whole cohort** (n=765) | | |  |
|  |  | (n) | OR | P | (n) | OR | P | (n) | OR | P | (n) | OR | P | (n) |
|  |  |  | *(CI;95%)* |  |  | *(CI;95%)* |  |  | *(CI;95%)* |  |  | *(CI;95%)* |  |  |
| **E4(-)** | **E2/E2** | 0 | na | na | 0 | na | na | 1 | 0.63  (0.07-5.04) | 1.00 | 1 | 0.21  (0.025-1.65) | 0.165 | 8 |
|  | **E2/E3** | 16 | 0.68  (0.39-1.17) | 0.30 | 23 | 0.89  (0.55-1.43) | 0.49 | 21 | 0.89  (0.54-1.47) | 0.715 | 60 | 0.84  (0.61-1.18) | 0.362 | 117 |
|  | **E2/E2**  **E2/E3** | 16 | 0.69  (0.39-1.19) | 0.20 | 23 | 0.83  (0.52-1.34) | 0.72 | 22 | 0.88  (0.54-1.42) | 0.635 | 61 | 0.80  (0.58-1.11) | 0.220 | 125 |
| **E4(+)** | **E2/E4** | 7 | 3.78  (1.41-10.10) | **0.011** | 6 | 2.72  (0.97-7.59) | 0.093 | 4 | 2.00  (0.62-6.46) | 0.272 | 17 | 2.81  (1.27-6.19) | **0.0091** | 10 |
|  | **E3/E4** | 93 | 4.22  (3.06-5.82) | **<0.0001** | 40 | 1.52  (1.03-2.26) | **0.038** | 40 | 1.68  (1.13-2.50) | **0.013** | 173 | 2.40  (1.85-3.11) | **<0.0001** | 119 |
|  | **E4/E4** | 7 | 7.56  (2.27-24.15) | **0.001** | 4 | 3.63  (0.69-13.67) | 0.063 | 3 | 3.00  (0.71-12.70) | 0.138 | 14 | 4.63  (1.65-12.93) | **0.0030** | 5 |
| **E3/E3**  (Reference) | | 153 | - | **-** | 182 | - | - | 165 | - | - | 500 | - | **-** | 827 |
| **E4(+)** | | 107 | 4.49  (3.32-6.08) | **<0.0001** | 50 | 1.73  (1.21-2.48) | **0.00033** | 47 | 1.78  (1.24-2.58) | **0.0032** | 204 | 2.58  (2.02-3.28) | **<0.0001** | 134 |
| **E4(-)**  (Reference) | | 169 |  |  | 205 |  |  | 187 |  |  | 561 |  |  | 952 |

Upper Table: ORs were obtained comparing each *APOE* genotype with *APO*E3/E3 as reference group; na, not applicable. Lower Table: absence (E4-) or presence (E4+) of the *APOE*4-allele was the unique considered discriminant for ORs calculation. Significant *P*≤0.05 are shown in bold.
