# Supplementary material for: Gene-gene interactions among coding genes of iron-homeostasis proteins and APOE-alleles in cognitive impairment diseases
Source: PLoS One. 2018 Mar 8;13(3):e0193867. doi: 10.1371/journal.pone.0193867 (PMC5843269; doi:10.1371/journal.pone.0193867)
Supplement: S3 Table — (DOCX) [file pone.0193867.s003.docx]

**S3 Table. Mini mental state examination (MMSE) in patients stratified by type of dementia and SNPs genotypes.**

|  | ***HFE* C282Y** | | ***HFE* H63D** | | ***FPN1* -8CG** | | ***HAMP* -582AG** | | ***TF* P570S** | | ***APOE**** | |
| --- | --- | --- | --- | --- | --- | --- | --- | --- | --- | --- | --- | --- |
|  |  | *MMSE* |  | *MMSE* |  | *MMSE* |  | *MMSE* |  | *MMSE* |  | *MMSE* |
| **AD**  **(n=276)** | CC | 19.7  (16.4-23.0) | HH | 19.7  (16.6-23) | CC | 20.0  (16.1-23.0) | AA | 20.3  (16.9-23.3) | PP | 19.7  (16.7-22.4) | E4(-) | 20.4  (16.5-23.4) |
|  | CY | 18.5  (15.6-19.8) | HD | 19.2  (15.9-22.4) | CG | 19.7  (17.2-22.4) | AG | 18.7  (15.7-22.1) | PS | 19.4  (15.7-23.4) | E4(+) | 18.7  (15.8-21.3) |
|  | - | - | DD | 17.9  (14.8-20.8) | GG | 17.8  (11.7-21.1) | GG | 20.5  (15.3-22.8) | SS | 19.1  (14.0-23.1) |  |  |
| *P* |  | 0.074 |  | 0.17 |  | 0.062 |  | 0.44 |  | 0.35 |  | **0.038** |
| **VaD**  **(n=255)** | CC | 19.7  (16.4-21.7) | HH | 19.4  (16.0-21.7) | CC | 20.0  (16.4-21.7) | AA | 19.1  (16.4-21.7) | PP | 20.0  (16.7-21.7) | E4(-) | 19.4  (16.2-21.7) |
|  | CY | 18.6  (18.5-20.4) | HD | 19.7  (16.7-21.5) | CG | 19.3  (16.3-21.8) | AG | 20.0  (17.0-21.5) | PS | 19.0  (15.1-21.6) | E4(+) | 19.8  (17.4-21.5) |
|  | - | - | DD | 19.8  (18.9-22.4) | GG | 16.7  (13.0-19.3) | GG | 20.5  (13.7-22.7) | SS | 17.8  (14.5-23.6) |  |  |
| *P* |  | 0.33 |  | 0.099 |  | 0.08 |  | 0.34 |  | 0.43 |  | 0.22 |
| **MCI**  **(n=234)** | CC | 25.4  (23.6-26.8) | HH | 25.4  (23.5-27) | CC | 25.0  (23.4-27.0) | AA | 25.3  (23.3-26.7) | PP | 25.0  (23.3-26.7) | E4(-) | 25.3  (23.6-26.8) |
|  | CY | 25.3  (23.6-26.7) | HD | 24.7  (23.4-26.7) | CG | 25.7  (23.7-26.7) | AG | 25.0  (23.6-26.7) | PS | 25.3  (24.0-27.0) | E4(+) | 24.2  (22.85-26.5) |
|  | - | - | DD | 25.0  (24.6-25.7) | GG | 25.2  (22.9-26.7) | GG | 25.6  (24.4-26.8) | SS | 24.0  (23.5-26.7) |  |  |
| *P* |  | 0.40 |  | 0.73 |  | 0.59 |  | 0.14 |  | 0.45 |  | 0.26 |
| **Whole**  **Cohort**  **(n=765)** | CC | 21.4  (17.7-24.5) | HH | 21.4  (17.8-24.5) | CC | 21.3  (17.7-24.2) | AA | 21.5  (17.7-24.5) | PP | 21.4  (18.1-24.4) | E4(-) | 21.7  (18.0-25.0) |
|  | CY | 18.7  (18.4-21.0) | HD | 21.3  (17.4-24.2) | CG | 21.8  (18.2-25.0) | AG | 21.0  (17.9-24.3) | PS | 21.0  (17.2-24.7) | E4(+) | 20.5  (17.4-23.5) |
|  | - | - | DD | 20.2  (18.0-23.9) | GG | 21.5  (16.8-24.9) | GG | 22.4  (15.8-24.5) | SS | 23.0  (15.7-24.4) |  |  |
| *P* |  | 0.11 |  | 0.27 |  | 0.28 |  | 0.32 |  | 0.28 |  | **0.018** |

MMSE was expressed as median and interquartile range and comparisons were performed comparing homozygotes for the polymorphic allele with the rest of genotypes with the exception of *HFE* C282Y in which homozygotes for the common allele (CC) were computed *versus* heterozygotes (CY). *, absence (E4-) or presence (E4+) of the *APOE*4-allele was the unique considered discriminant for *P* calculation. Significant *P*≤0.05 are shown in bold.
