# Supplementary material for: Gene-gene interactions among coding genes of iron-homeostasis proteins and APOE-alleles in cognitive impairment diseases
Source: PLoS One. 2018 Mar 8;13(3):e0193867. doi: 10.1371/journal.pone.0193867 (PMC5843269; doi:10.1371/journal.pone.0193867)
Supplement: S4 Table — ORs evaluation comparing multicarriers (A) or no carriers (B) of polymorphic alleles in iron genes stratified by APOE4 condition. (DOCX) [file pone.0193867.s004.docx]

**S4 Table. ORs evaluation comparing multicarriers (A) or no carriers (B) of polymorphic alleles in iron genes stratified by *APOE*4 condition.**

| **A** | **At least 3 polymorphic alleles in iron genes** | | | | | | |
| --- | --- | --- | --- | --- | --- | --- | --- |
| **Cases** (n=765) | | | **Controls** (n=1086) | | **OR (CI;95%); *P*** | | |
| **Cognitive** | | **Stratified by** | **(n)** | **Stratified by** | **Cognitive**  **diagnosis** | **OR^1^** | **OR^2^** |
| **diagnosis (n)** | | ***APOE*4 condition**  **(n)** |  | ***APOE*4 condition (n)** |  |  |  |
| **AD** | | *APOE*4(+) n=22 | (218) | *APOE*4(+) | **AD** | 0.99 (0.71-1.07) | 0.65 (0.43-0.97) |
| (55) | | *APOE*4(-) n=33 |  |  |  | n.s. | **0.035** |
| **VaD** | | *APOE*4(+) n=11 |  | (31) | **VaD** | 0.9 (0.63-1.28) | 0.76 (0.51-1.12) |
| (46) | | *APOE*4(-) n=35 |  |  |  | n.s. | n.s. |
| **MCI** | | *APOE*4(+) n=4 |  | *APOE*4(-) | **MCI** | 0.79 (0.55-1.16) | 0.84 (0.57-1.25) |
| (39) | | *APOE*4(-) n=35 |  |  |  | n.s. | n.s. |
| **Whole cohort** | | *APOE*4(+) n=37 |  | (187) | **Whole**  **cohort** | 0.89 (0.7-1.13) | 0.74 (0.57-0.97) |
| (140) | | *APOE*4(-) n=103 |  |  |  | n.s. | **0.030** |
| **B** | **No polymorphic alleles in iron genes** | | | | | | |
| **Cases** (n=765) | | | **Controls** (n=1086) | | **OR (CI;95%); *P*** | | |
| **Cognitive**  **diagnosis (n)** | | **Stratified by** | **(n)** | **Stratified by** | **Cognitive**  **diagnosis** | **OR^1^** | **OR^3^** |
|  |  | ***APOE*4 condition**  **(n)** |  | ***APOE*4 condition (n)** |  |  |  |
| **AD** | | *APOE*4(+) n=23 | (153) | *APOE*4(+) | **AD** | 1.52 (1.08-2.13) | 5.11 (2.74-5.52) |
| (55) | | *APOE*4(-) n=32 |  |  |  | **0.019** | **<0.0001** |
| **VaD** | | *APOE*4(+) n=9 |  | (19) | **VaD** | 1.43 (1.05-2.12) | 2.05 (0.92-4.6) |
| (50) | | *APOE*4(-) n=41 |  |  |  | **0.032** | 0.08 |
| **MCI** | | *APOE*4(+) n=9 |  | *APOE*4(-) | **MCI** | 1.30 (0.89-1.9) | 2.24 (1.0-5.02) |
| (41) | | *APOE*4(-) n=32 |  |  |  | n.s. | **0.05** |
| **Whole cohort** | | *APOE*4(+) n=41 |  | (134) | **Whole**  **cohort** | 1.44 (1.12-1.85) | 3.18 (1.83-5.52) |
| (146) | | *APOE*4(-) n=105 |  |  |  | **0.0047** | **<0.0001** |

OR^1^, OR obtained comparing cases and controls regardless *APOE* condition;

OR^2^, OR obtained comparing cases and controls negative for *APOE*4 allele;

OR^3^, OR obtained comparing cases and controls positive for *APOE*4 allele.
