# Supplementary material for: Gene-gene interactions among coding genes of iron-homeostasis proteins and APOE-alleles in cognitive impairment diseases
Source: PLoS One. 2018 Mar 8;13(3):e0193867. doi: 10.1371/journal.pone.0193867 (PMC5843269; doi:10.1371/journal.pone.0193867)
Supplement: S5 Table — MMSE comparison in patients multicarrier (A) or no carriers (B) of polymorphic alleles in iron genes stratified by APOE4 condition. (DOCX) [file pone.0193867.s005.docx]

**S5 Table. MMSE comparison in patients multicarrier (A) or no carriers (B) of polymorphic alleles in iron genes stratified by *APOE*4 condition.**

| **A** | **At least 3 polymorphic alleles in iron genes** | | | | **B** | **No polymorphic alleles in iron genes** | | | |
| --- | --- | --- | --- | --- | --- | --- | --- | --- | --- |
| **Cognitive diagnosis**  (n) | **MMSE**  Median (interquartile range) | | | | **Cognitive diagnosis**  (n) | **MMSE**  Median (interquartile range) | | | |
|  | MMSE Regardless  to *APOE* condition | *APOE*4 condition | MMSE  stratified by *APOE*4 condition | *P* |  | MMSE Regardless  to *APOE* condition | *APOE*4  condition | MMSE  stratified by *APOE*4 condition | *P* |
| **AD**  (55) | 18.7  (15.3-22.5) | *APOE*4(+) n=22 | 17.4  (15.4-19.9) | 0.13 | **AD**  (55) | 20.2  (17.6 -22.9) | *APOE*4(+) n=23 | 19.0  (17.6-22.2) | 0.49 |
|  |  | *APOE*4(-) n=33 | 19.9  (13.2-24.0) |  |  |  | *APOE*4(-) n=32 | 21.0  (17.6-22.9) |  |
| **VaD**  (46) | 19.7  (16.2-22.4) | *APOE*4(+) n=11 | 19.7  (16.0-22.0) | 0.37 | **VaD**  (50) | 20.0  (16.5-22.0) | *APOE*4(+) n=9 | 21.3  (19.0-22.0) | 0.073 |
|  |  | *APOE*4(-) n=35 | 19.7  (16.3-22.1) |  |  |  | *APOE*4(-) n=41 | 20.0  (16.0-22.0) |  |
| **MCI**  (39) | 25.0  (24.0-26.7) | *APOE*4(+) n=4 | 24.5  (23.5 -25.5) | 0.30 | **MCI**  (41) | 25.0  (22.7-27) | *APOE*4(+) n=9 | 24.7  (22.7-25.9) | 0.27 |
|  |  | *APOE*4(-) n=35 | 25.3  (24.0-26.7) |  |  |  | *APOE*4(-) n=32 | 25.0  (23.0-27.1) |  |
| **Whole**  **cohort**  (140) | 21.0  (17.4-24.4) | *APOE*4(+) n=37 | 18.5  (15.7 -22.4) | **0.006** | **Whole cohort**  (146) | 21.4  (18.5 -23.7) | *APOE*4(+) n=41 | 21.0  (18.7-23.4) | 0.38 |
|  |  | *APOE*4(-) n=103 | 22.4  (18.3-24.1) |  |  |  | *APOE*4(-) n=105 | 21.7  (18.5-24.4) |  |
