# Supplementary material for: Gene-gene interactions among coding genes of iron-homeostasis proteins and APOE-alleles in cognitive impairment diseases
Source: PLoS One. 2018 Mar 8;13(3):e0193867. doi: 10.1371/journal.pone.0193867 (PMC5843269; doi:10.1371/journal.pone.0193867)
Supplement: S6 Table — (DOCX) [file pone.0193867.s006.docx]

**S6 Table. Summary ORs from selected meta-analyses on AD and HFE and TF SNPs.**

| **Meta-analyses** | **HFE C282Y** | | **HFE H63D** | | **TF P570S** | |
| --- | --- | --- | --- | --- | --- | --- |
|  | *Genetic model* | *ORs* | *Genetic model* | *ORs* | *Genetic model* | *ORs* |
| Lin et al, 2012  [37] | Y *vs* C | 1.004 [0.89-1.13] | D *vs* H | 0.902 [0.82-0.99] | - | - |
|  | YY+YC *vs* CC | 1.04 [0.91-1.18] | DD+DH  *vs* HH | 0.89 [0.79-0.99] | - | - |
|  | YY *vs* YC+CC | 0.86 [0.48-1.54] | DD *vs* DH+HH | 0.94 [0.7-1.26] |  |  |
| Wang et al, 2013  [53] | - | - | - | - | S *vs* P | 1.13 [1.06-1.21] |
|  | - | - | - | - | SS+SP *vs* PP | 1.11 [1.05-1.17] |
|  | - | - | - | - | SS *vs* SP+PP | 1.23 [1.03-1.47] |
| AlzGene  Forum * | Y *vs* C | 1.04 [0.88-1.23] | D *vs* H | 0.90 [0.74-1.08] | S *vs* P | 1.18 [1.06-1.31] |

*: Source <https://www.alzforum.org/> For details on specific ethnicity or for additional genetic models considered in the studies see the cited references.
